# Effects of essential metals (iron, zinc, and copper) on thyroid diseases - a narrative review

**DOI:** 10.3389/fmed.2026.1786788

**Published:** 2026-04-10

**Authors:** Hongxia Wei, Zhe Li, Zi'ang Liu, Baofeng Wu, Ru Li, Ming Xu, Xifeng Yang, Jianhong Yin, Yi Zhang, Yunfeng Liu

**Affiliations:** 1 Department of Endocrinology, The First Hospital of Shanxi Medical University, Taiyuan, China; 2 The First Clinical Medical College, Shanxi Medical University, Taiyuan, China; 3 Department of Pharmacology, Shanxi Medical University, Taiyuan, China; 4 Medicinal Basic Research Innovation Center of Chronic Kidney Disease, Ministry of Education, Shanxi Medical University, Taiyuan, China; 5 Clinical Research Center for Endocrine and Metabolic Diseases of Shanxi Medical University, Taiyuan, China

**Keywords:** autoimmune thyroid diseases, copper, hypothyroidism, iron, thyroid cancer, zinc

## Abstract

The thyroid gland is the largest endocrine organ in the human body, and alterations in its homeostasis may lead to the development of thyroid diseases. The role of essential metals such as iron (Fe), zinc (Zn), and copper (Cu) in the pathogenesis of thyroid diseases has been widely discussed, but the research results are inconsistent. In addition, the therapeutic role of these essential metal elements in patients with thyroid diseases has been significantly underestimated in the clinical practice. A disturbance in the balance of these essential metals will affect thyroid homeostasis in a variety of ways. This article synthesizes current evidence on the effects of essential metal elements (Fe, Zn, and Cu) on a variety of thyroid diseases, such as hypothyroidism, autoimmune thyroid diseases (AITD), and thyroid cancer. It will provide essential metal element-related clues for the development of therapeutic strategies and pathophysiologic studies of thyroid diseases.

## 1 Introduction

As the largest endocrine organ in the body, the thyroid gland produces and secretes thyroid hormones (THs), such as triiodothyronine (T3) and thyroxine (T4) (1). THs can affect the development and maintenance of almost any organ or system. Growth and development, oxidative metabolism, and regulating basal metabolic rate are all affected by THs (1).Thyroid disease is a common endocrine disorder, including hypothyroidism, autoimmune thyroid diseases (AITD), and thyroid cancer, etc, with an increasing incidence in recent years (2–4). AITD is an autoimmune thyroid disease, with the most common diseases being Hashimoto's thyroiditis (HT) and Grave's disease (GD) (5). HT is the most common cause of hypothyroidism, and the serological markers of HT are thyroid peroxidase antibody (TPOAb) and thyroglobulin antibody (TGAb) (6). GD is characterized by the production of stimulating autoantibodies to the thyroid-stimulating hormone receptor, which can lead to hyperthyroidism (7).

The critical role of essential metal elements such as iron (Fe), zinc (Zn), and copper (Cu) in thyroid pathophysiology has sparked global interest. Deficiency or excess of these essential metals can disrupt THs homeostasis, which in turn impacts the metabolism of essential metal elements (8). Although the concentration of essential metal elements in the human body is very low, they play a vital role in human health. These essential metals act as catalytic and structural cofactors for key proteins involved in various biochemical processes, including energy metabolism, antioxidant metabolism, nucleic acid and protein synthesis, immune function, gene expression, and cell apoptosis (9–12). Therefore, alterations in the levels and distribution of essential metals in both animals and humans may result in a range of pathophysiological conditions, potentially contributing to the onset of thyroid diseases. There are variations in the levels of essential metals in serum, plasma, or thyroid tissue among different populations (13–15), which may have differential effects on thyroid metabolism.

Although numerous studies have investigated the association between essential metals and thyroid diseases, the findings remain inconsistent, and the specific mechanisms by which essential metal elements such as Fe, Zn, and Cu affect thyroid function remain incompletely understood. This review provides a comprehensive review of the relationship between essential metal elements (Fe, Zn, and Cu) and thyroid diseases, aiming to establish a theoretical foundation related to trace essential metal elements for the investigation of the pathogenesis and treatment strategies of thyroid diseases.

## 2 Methods

Relevant literature was sourced from the PubMed and Embase databases.For PubMed specifically, our search approach merged MeSH with free-text keywords, with all terms combined through the use of Boolean operators:

(“Iron”[Mesh] OR “Zinc”[Mesh] OR “Copper”[Mesh] OR “iron” OR “fe” OR “zinc” OR “zn” OR “copper” OR “cu”) AND ((“Thyroid Diseases”[Mesh]) OR (“Hypothyroidism”[Mesh] OR “thyroid stimulating hormone deficiency”) OR (“Thyroiditis, Autoimmune”[Mesh] OR “autoimmune thyroiditis” OR “autoimmune thyroid disease” OR “hashimoto's thyroiditis” OR “hashimoto disease”) OR (“Graves Disease”[Mesh] OR “hyperthyroidism, autoimmune” OR “exophthalmic goiter” OR “basedow disease”) OR (“Graves Ophthalmopathy”[Mesh] OR “Graves orbitopathy” OR “dysthyroid ophthalmopathy” OR “Graves eye disease”) OR (“thyroid neoplasms”[Mesh] OR “thyroid carcinoma” OR “thyroid cancer” OR “thyroid adenoma”)).

The search primarily focused on studies published published between 2010 and 2025. In addition, classic and landmark publications prior to 2010 that remain widely cited and foundational, as well as earlier studies on topics with limited recent research, were also included. We performed a supplementary search in Embase using an analogous search strategy. Inclusion was restricted to English-language original research, reviews, and meta-analyses. The initial search yielded 694 records; after deduplication, manual screening of titles and abstracts, and a hand-search of reference lists, we ultimately retained 153 articles for narrative synthesis.

## 3 Iron and thyroid diseases

Fe primarily exists in the form of Fe^2+^ and Fe^3+^, and participates in the composition of functional proteins such as hemoglobin, myoglobin, transferrin and other functional proteins. Furthermore, it exists in the form of stored Fe, such as ferritin and hemosiderin. In addition, it also plays a crucial role in maintaining the activity of various Fe-containing and Fe-dependent enzymes. Iron deficiency (ID) represents the primary etiological factor contributing to the development of anemia. Serum ferritin, a complex of Fe ions and Apoferritin, is responsible for storing and buffering excess Fe, and is the most sensitive indicator of ID, therefore, it has a good diagnostic and predictive value for ID (16). Fe is indispensable for proper cellular function as it facilitates oxygen and electron transfer while playing a critical role in numerous enzyme activities. Approximately 2% of human genes encode Fe-binding proteins, with 6.5% of enzymes directly dependent on Fe (16).

### 3.1 Iron and hypothyroidism

#### 3.1.1 Iron deficiency and hypothyroidism

##### 3.1.1.1 Clinical study on the relationship between iron and hypothyroidism

ID exerts a significant impact on thyroid function. A cross-sectional study based on a nationwide general adult population in Spain has confirmed that ID (defined as a serum ferritin concentration < 30 μg/L) is independently associated with an increased risk of hypothyroxinemia and hypotriiodothyroninemia, and this correlation remains consistent regardless of sex, menstrual status, or iodine nutritional status (17). In addition, multiple studies have established an association between gestational ID and hypothyroidism, which is elaborated in detail in Section 3.4. For adult patients with subclinical or overt primary hypothyroidism complicated by ID, Fe supplementation serves an important adjunctive role. In the study by Ravanbod et al. (18), patients with iron deficiency anemia (IDA) and concurrent subclinical hypothyroidism exhibited no significant changes in Hb and thyroid stimulating hormone (TSH) levels after 90 days of monotherapy with either Fe or levothyroxine (L-T4). However, the group receiving combined Fe and L-T4 therapy showed a significant elevation in Hb and serum ferritin levels, alongside a significant reduction in TSH. These findings demonstrate that combination therapy with L-T4 and Fe is effective for patients with IDA combined with subclinical hypothyroidism. Rayman et al. (19) recommended that patients with hypothyroidism undergo routine screening for ID. If ID is confirmed or serum ferritin levels are < 70 μg/L, Fe supplementation should be initiated concurrently with management of the underlying cause of ID, to mitigate the adverse effects of ID on thyroid function. Therefore, for adult patients with primary subclinical or overt hypothyroidism who have either a serum ferritin level < 70 μg/L or concomitant ID / IDA, combination therapy with L-T4 and Fe preparations can effectively improve their thyroid function. It is recommended that Fe-containing preparations be administered at least 4 h apart from L-T4 to avoid impaired L-T4 absorption (20). This interaction occurs because iron salts can form insoluble complexes with the phenol, carboxylic acid, and amine functional groups of L-T4, thereby reducing the drug's bioavailability (21).

##### 3.1.1.2 Mechanisms of iron deficiency affecting hypothyroidism

**Figure 1:**
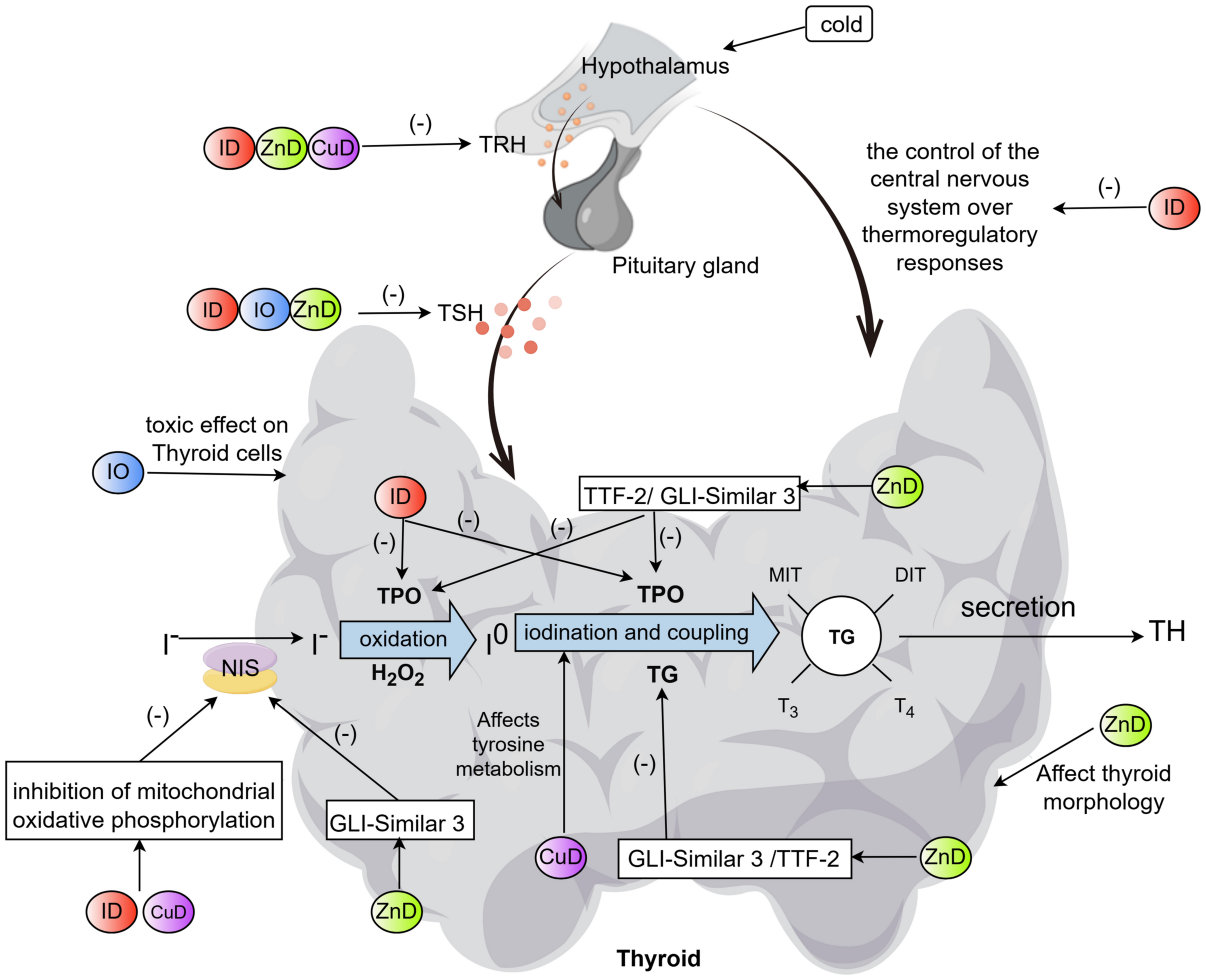
Effects of three essential metal elements (Fe, Zn, and Cu) on HPT axis (By Figdraw). Specifically, these include: (1) ID inhibits the HPT axis by suppressing TRH secretion and attenuating the response of TSH to TRH. ID reduces TPO activity (affecting iodide oxidation, iodination, and coupling processes) and decreases NIS-mediated iodide uptake by inhibiting mitochondrial oxidative phosphorylation. ID also blunts the TH response to cold exposure by suppressing the central nervous system's control over thermoregulatory responses. (2) IO induces cytotoxicity in thyroid cells and reduces pituitary TSH secretion. (3) ZnD inhibits the HPT axis (decreasing the production and release of TRH and TSH, as well as the response of TSH to TRH) and impairs the functions of transcription factors TTF-2 and GLI-Similar 3. This leads to reduced transcription of NIS, TPO, and TG, ultimately decreasing TH synthesis. ZnD also alters thyroid morphology and volume. (4) CuD reduces TRH activity, inhibits mitochondrial oxidative phosphorylation to decrease NIS-mediated iodide uptake, and interferes with tyrosine metabolism, thereby hindering THs synthesis. (+) denotes promotion/activation, and (–) indicates inhibition/reduction. NIS, sodium/iodide symporter; ID, iron deficiency; IO, iron overload; ZnD, zinc deficiency; CuD, copper deficiency; TRH, thyrotropin-releasing hormone; TSH, thyroid stimulating hormone; TH, thyroid hormone; TG, thyroglobulin; TPO, thyroid peroxidase; MIT, monoiodotyrosine; DIT, diiodotyrosine; T_3_, triiodothyronine; T_4_, throxine; TTF-2, Thyroid transcription factor 2.

**Figure 2:**
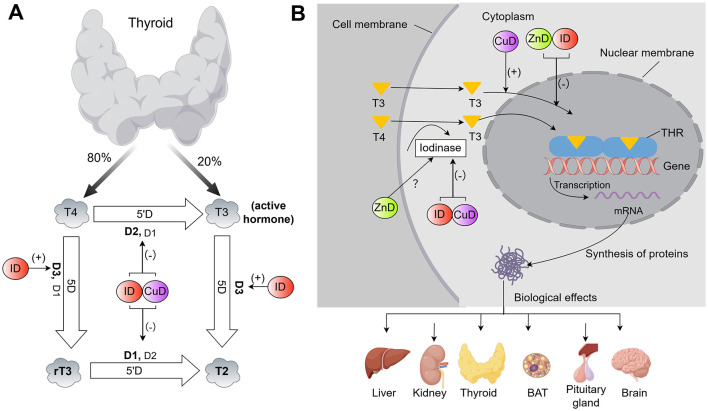
Effects of three essential metal elements (Fe, Zn and Cu) on **(A)** peripheral deiodination of THs and **(B)** target cell effects of THs (By Figdraw). Specifically, these effects are as follows: (1) In the peripheral pathway, ID and CuD suppress D1 and D2 activities, thereby reducing both systemic and local T4-to-T3 conversion. ID also enhances D3 activity, accelerating the clearance of T3 and T4. The precise impact of ZnD on iodothyronine deiodinase activity remains unclear. (2) Within target cells, ID and ZnD inhibit T3 binding to its nuclear receptor and downregulate subsequent gene transcription. This disruption alters target protein synthesis and blunts the biological effects of THs across multiple organs. However, the opposite effect is observed in the context of CuD, which may represent an adaptive cellular response. Note: (+) denotes promotion/activation, (–) indicates inhibition/reduction, and “?” indicates an unclear or unconfirmed regulatory relationship. ID, iron deficiency; IO, iron overload; ZnD, zinc deficiency; CuD, copper deficiency; T3, triiodothyronine; T4, thyroxine; rT3, reverse T3; T2, 3,3′-diiodothyronine; D1, D2, D3, type I-III deiodinase; 5′D, 5′-deiodination (outer ring deiodination); 5D, 5-deiodination (inner ring deiodination); THR, thyroid hormone receptor.

By affecting the hypothalamic-pituitary-thyroid (HPT) axis, the activity of thyroxine deiodinase and Fe-containing enzymes, as well as oxygen transport, ID reduces the synthesis of THs or the conversion rate of peripheral T3 (Figures 1, 2).

ID can affect the HPT axis (Figure 1). An animal study found that plasma thyroid-stimulating hormone (TSH) showed a blunted response to exogenous thyrotropin-releasing hormone (TRH) administration in IDA rats, leading to decreased secretion of THs (22). It was revealed in rat animal models that ID regulates thyroid metabolism by indirectly altering the central nervous system's control over thermoregulatory responses (23). During cold exposure, sympathetic nerve endings release more norepinephrine, which acts on adrenergic receptors in brown adipose tissue, thereby enhancing thermogenesis (23). The activation of adrenergic receptors stimulates the synthesis of 5′-deiodination deiodinase messenger RNA (mRNA), increasing the concentration of T3 in brown adipose tissue and saturating nuclear T3 receptors. Adrenergic-dependent signals act together with nuclear T3 receptor saturation to induce uncoupling protein mRNA synthesis. This protein is responsible for brown adipose tissue thermogenesis. Therefore, in rats, cold environments are expected to increase TH levels and turnover rate, but these processes are weakened in the ID state (23). Furthermore, the sluggish response of ID to cold may be attributed to dysfunction within the HPT axis (24). Tang et al. (25) suggested that the inability of ID rats to respond to cold may stem from a reduction in TRH released from the hypothalamus. The inhibition of TRH secretion in the ID state may be attributed to an increased release of dopamine within the ventral neuronal pathways of the midbrain (23).

*In vivo*, ID leads to decreased activity of Fe-dependent enzymes and Fe-containing enzymes (Figure 1). Thyroid peroxidase (TPO) is a heme-dependent enzyme, and the porphyrin ring center of heme is a Fe^2+^. TPO plays an important role in catalyzing two key steps in THs synthesis: the iodination of thyroglobulin (TG) and the coupling of iodine tyrosine molecules (26). ID leads to decreased TPO activity, which impairs iodine activation, iodination of tyrosine residues and coupling to THs, resulting in reduced THs synthesis and increased TSH in rat (27). In addition, the enzymes of the tricarboxylic acid cycle and the electron transport system require Fe as a cofactor. ID reduces the activity of these enzymes, resulting in reduced adenosine triphosphate (ATP) formation, iodine uptake capacity of thyroid follicular cells and synthesis of THs (28).

ID can interfere with the activity of thyronine deiodinase (Figure 2). There are three types of thyronine deiodinases: type I-III deiodinase (D1-D3). D1 and D2 can convert T4 to T3 through 5′-deiodination (outer ring deiodination), maintaining circulating and local T3 concentrations, respectively. D3 mainly converts T4 to reverse T3 with almost no physiological activity, or degrades T3 to 3,3′-T2 through 5-deiodination (inner ring deiodination) (22, 29). Most of the effects of THs are mainly generated by T3 with high biological activity, so the role of 5′-deiodination in THs onset of action is very important (30). Kidney, liver and thyroid have the highest D1 activity, while pituitary gland, brain and brown adipose tissue have the highest D2 activity (30). In animals with hypothyroidism, pituitary D2 activity is increased, mainly providing T3 for intracellular utilization; In contrast, D1 activity in the liver, kidney, and muscle is decreased (22). ID reduces the conversion of T4 to T3 by interfering with TH deiodinase activity in rats (23). Beard et al. (22) found that the turnover rate of T3 was significantly lower in IDA rats than in control rats (42 ng/h vs. 88 ng/h), as well as the total activities of liver D1 and D2. ID can also decrease D1 and D2 activity and increase D3 activity in the human body, so that the T3 production is reduced and the T3 and T4 clearance rate is increased, which leads to hypothyroidism (31). This may be a protective response of the organism in the ID state.

The action of THs is mediated by the binding of T3 to the nuclear TH receptors (32). Animal studies indicate that ID can inhibit the binding of T3 to its nuclear receptor, resulting in slower utilization of T3 in the serum pool (33) (Figure 2B). ID can also interfere with thyroid metabolism by affecting the synthesis of hemoglobin, leading to a reduced oxygen-carrying capacity of the blood. This is similar to thyroid damage during hypoxia, which results in greatly reduced iodine uptake, TH biosynthesis, and iodine release rate (34).

#### 3.1.2 Iron overload and hypothyroidism

Patients with β-thalassemia suffer from iron overload (IO) due to the need for long-term chronic blood transfusions, and this iron-toxic injury can lead to hypothyroidism (especially primary hypothyroidism). Patients with serum ferritin levels higher than 1,500 μg/L have a progressive development of hypothyroidism (35). When Fe chelation therapy is administered late or the patient is poorly adherent, hypothyroidism begins earlier (36). Serum ferritin is a simple and reliable indicator of Fe loading (37). A meta-analysis showed that β-thalassemia patients with hypothyroidism had significantly higher serum ferritin levels compared to β-thalassemia patients with normal thyroid function, which suggested a significant association between Fe loading and hypothyroidism (38).

IO can cause Fe deposition in the thyroid parenchyma and stromal cells, resulting in thyroid hemochromatosis, which leads to progressive deterioration of thyroid function (39). IO also leads to transferrin saturation, at which point non-transferrin-bound Fe appears in plasma as a toxic form of Fe. Free iron in non-transferrin-bound can induce the production of Reactive Oxygen Species (ROS) through the Fenton reaction (Fe^2+^+H2O2 → Fe^3+^+•OH + OH–). ROS can cause lipid peroxidation, resulting in cellular loss of function and cellular death (one of the classical regulatory pathways of cellular ferroptosis) (40). ROS is highly toxic to thyroid (especially the anterior lobe of the thyroid) cells (41). Under IO conditions, lipid peroxidation markers and unsaturated and saturated aldehydes (e.g., malondialdehyde, hexanal) are produced, both of which are associated with cellular dysfunction, cytotoxicity, and cell death (36). The incidence of hypothyroidism is increased in β-thalassemia major patients with splenectomy (42) because the spleen may have a potential clearance effect on free Fe, including non-transferrin-bound Fe. The buffering capacity of extrahepatic Fe is reduced after splenectomy (43). On the other hand, *in vitro* studies have shown that TRH plays an important role in stimulating the activity of TSHβ promoter by activating protein kinase C (44). Protein kinase C is regulated by Fe (45), and IO may have a detrimental effect on its function, resulting in insufficient pituitary TSH secretion (Figure 1). However, a clinical observational study found that IO-induced injury had little effect on the HPT axis, and that thyroid function had already failed before the HPT axis was impaired (46).

### 3.2 Iron and AITD

#### 3.2.1 Iron deficiency and AITD

Several studies have indicated a strong association between ID and AITD, although the precise mechanism remains unclear. A meta-analysis showed that Fe concentrations were reduced in a subgroup of patients with HT compared to healthy controls (47). A cross-sectional study conducted by Erdal et al. (48) in an iodine-sufficient region of Turkey also yielded consistent findings. Luo et al. (49) found that after adjusting for confounding factors including age, body mass index, smoking, alcohol consumption, and iodine nutritional status, serum Fe level was significantly negatively correlated with serum TPOAb and TGAb levels, especially in women of childbearing age, and serum Fe level had a greater impact on TPOAb positivity than TGAb. A cross-sectional study conducted by Zhang et al. (50) in an iodine-sufficient region of China confirmed that after adjusting for relevant confounders, ID in women of childbearing age was still associated with higher TPOAb positivity, but not with TGAb. The authors explain this phenomenon by the fact that TPO undergoes post-translational modification due to ID, exposing previously hidden epitopes or generating new epitopes, thereby enhancing the immunogenicity of TPO (50). However, there is currently no sufficient clinical evidence to support that Fe supplementation can improve thyroid function in patients with AITD.

Fe is essential for Deoxyribonucleic Acid (DNA) synthesis. ID can lead to impaired DNA synthesis and interfere with the biological production and normal expression of microRNA (33). Through the influence of DNA repair mechanisms and Fe on complex epigenetic interactions, ID can induce changes in the genome, which may promote the development of AITD (33). In addition, ID can promote oxidative stress, manifested by elevated levels of oxidants and/or decreased capacity of antioxidant enzymes, which also plays a non-negligible role in the pathogenesis of AITD.

The bio-utilization of Fe controls complex metabolic programs in immune cell homeostasis and inflammation (51). ID can lead to disruption of immune tolerance and dysfunction of cellular and humoral immunity, which may be a possible mechanism leading to the development of AITD. ID can lead to dysfunction of the adaptive immune response, which promotes the production of antithyroid antibodies by intrathyroidal B lymphocytes (33). Fe in the plasma modulates innate immunity by regulating the ratio of monocytes to neutrophils and neutrophil activity. Hypoferremia inhibits the antibacterial function of neutrophils but enhances mitochondrial ROS-mediated NETosis (neutrophil extracellular traps), inducing chronic inflammation (52).IFN-γ inhibits the production of ferritin and decreases the density of the transferrin receptor under lipopolysaccharide induction, and administration of IL-4 before lipopolysaccharide induction reverses this phenomenon. ID can lead to an increase in IFN-γ producing lymphocytes and a decrease in IL-4 producing lymphocytes (53). Jiang et al. (54) demonstrated the critical role of Fe-dependent H3K9 demethylation in B-cell proliferation, establishing a link between Fe and humoral immunity. H3K9 demethylation is also essential for T cell differentiation, and ID may affect T lymphocyte differentiation (54). ID mice showed decreased activity of protein kinase C, an enzyme that plays a key role in T lymphocyte proliferation (55). In addition, ID can reduce the activity of other heme-containing enzymes, such as myeloperoxidase, so ID may trigger the production of antibodies against myeloperoxidase, which can cross-react with TPO, thus increasing the prevalence of AITD in patients (56).

#### 3.2.2 Iron overload and AITD

IO can cause a series of immune dysfunctions, such as altering the function of macrophages, monocytes, T cells and B cells and inhibiting the function of the complement system (57). Thyroid damage caused by Fe deposition may result in exposure to cellular antigens (e.g., TPO and TG), which stimulates the production of thyroid autoantibodies (58). However, there are few studies on the relationship between IO and AITD, and the conclusions are inconsistent. Zhang et al. (50) found that positivity for either TPOAb or TGAb alone was not associated with IO in pregnant women or non-pregnant women of childbearing age. Pes et al. (59) found that in β-thalassemia patients, the level of serum ferritin in TPOAb-positive patients was significantly higher than that in TPOAb-negative patients, and it is highly likely that the elevated level of TPOAb is an indicator of Fe-mediated tissue damage.

### 3.3 Iron and thyroid cancer

Fe is one of the drivers of the carcinogenic program. Fe catalyzes the formation of free radicals through the Fenton reaction, which causes DNA fragmentation, protein inactivation, and lipid peroxidation (60). In addition, Fe has the ability to catalyze the formation of DNA strand breaks and oxidize DNA bases (61), this makes Fe a potential mutagen that can induce cancer. It is well known that hemochromatosis is a genetic disorder characterized by Fe overload that increases the risk of developing hepatocellular carcinoma in patients (62). So, can Fe be used in the treatment of cancer (especially thyroid cancer)?

Because cancer cells have a strongly increased need for Fe to maintain high proliferation rates and tumor progression, ID (depriving cancer cells of Fe) has been explored as an anti-cancer strategy in preclinical studies. The study found that the high-iron diet promoted tumor growth in mice, while the iron-restricted diet or treatment with Fe chelators limited tumor growth (63–66). The dependence of tumor cells on Fe is enhanced, and the inhibitory effect of Fe restriction on the proliferation of cancer cells is greater than that of non-cancer cells. We call this phenomenon “Fe addiction” (67). Tumor growth can be inhibited by regulating proteins related to Fe metabolism, such as decreasing cellular Fe input by blocking transferrin, increasing cellular Fe output by overexpressing ferroportin, and disrupting Fe responsive element-binding proteins (68). Transferrin binds to Fe and is absorbed into cells through transferrin receptor mediated endocytosis. An *in vitro* study found that down-regulation of transferrin receptor 1 can inhibit the extracellular regulated protein kinases pathway in thyroid cancer cells, thus blocking the cell cycle and activating the apoptosis pathway (69). In addition, a study combining bioinformatics analysis with *in vitro* mechanistic investigations found that increased transferrin receptor 2 expression is associated with poor prognosis and proliferation of Papillary thyroid cancer (PTC) (70). Nuclear factor interleukin 3 contributes to the secretion of hepcidin in thyroid cancer cells by inhibiting the expression of Sclerostin domain-containing protein-1. Increased hepcidin leads to decreased expression of ferroportin and increased intracellular Fe levels, which promotes the proliferation of thyroid cancer cells. The absence of E4BP4 suppresses this effect (71).

Interestingly, the use of Fe to generate cytotoxic oxidative stress in cancer cells may hold potential for targeted cancer treatments (60). Ferroptosis is a recently discovered Fe-dependent regulated cell death mechanism characterized by the overwhelming, Fe-dependent accumulation of lethal lipid ROS (40). Ferroptosis inducers are preferentially cytotoxic to cancer cells (68). Numerous preclinical studies have demonstrated that inducing ferroptosis is a promising therapeutic treatment for thyroid cancer. Based on an analysis of the Cancer Genome Atlas and Gene Expression Omnibus databases, Chen et al. (72) demonstrated that glutathione peroxidase 4, a key ferroptosis-related regulator, is overexpressed in thyroid cancer, and that its upregulation is associated with T3–T4 tumor stages and pathological stage III–IV. Furthermore, patients with GPX4 overexpression exhibited a significantly poorer overall survival compared with those with low GPX4 expression (72).Functional assays *in vitro* and studies in *in vivo* animal models have demonstrated that two circular RNAs, Circ_0067934 and circKIF4A, can promote the proliferation of thyroid cancer cells by reducing ferroptosis (73, 74). Induction of ferroptosis, for example through the use of vitamin C or ALKBH5, has been shown to effectively inhibit the progression of thyroid cancer (75, 76). We also refer readers to a recent review that extensively summarizes current research on the role of ferroptosis in thyroid cancer, involving mechanisms, therapeutic targets, and predictive biomarkers (77).

Ultimately, the current data linking Fe to thyroid cancer rest almost entirely on preclinical models and retrospective bioinformatics, completely lacking validation in human cohorts. A glaring translational chasm separates these mechanistic insights from real-world clinical evidence, precluding any direct extrapolation to human applications. To bridge this gap, future research must urgently prioritize large-scale prospective cohorts and patient-centered interventional trials to rigorously test these preclinical hypotheses.

### 3.4 Effect of iron deficiency in pregnant women on thyroid function of self and offspring

ID is a prevalent nutritional concern during pregnancy. The prevalence of ID during the first trimester of pregnancy stood at approximately 37.0% (78). THs are critical for fetal neurological development during the first half of pregnancy, as the fetus does not begin to produce and secrete TH until 18–20 weeks of gestation (79). Mounting evidence links gestational ID to an elevated risk of maternal thyroid dysfunction (26, 28, 78–81). By rigorously controlling for urinary iodine and eliminating iodine supplementation confounders, a retrospective cohort by Teng et al. (79) (*n* = 723) established first- and second-trimester ID as an independent risk factor for gestational hypothyroxinemia. In a large cross-sectional study (*n* = 2,218), Wang et al. (28) demonstrated a significant association between gestational ID/IDA and altered thyroid function profiles—specifically reduced free triiodothyronine (FT3) and free thyroxine (FT4) alongside elevated TSH—and validated serum ferritin and hemoglobin as predictive markers for gestational FT4 levels. Despite its large sample size and granular stratified analyses, the study did not assess individual-level iodine nutritional status, relying exclusively on assumptions of regional iodine sufficiency. In a well-controlled cross-sectional analysis of 209 mid-pregnancy women (verified as urinary iodine-sufficient and autoantibody-negative), He et al. (31) identified serum ferritin as an independent determinant of TSH, detailing its negative relationship with TSH and positive link to FT4. While this study rigorously controlled for iodine-related confounding, its cross-sectional design and exclusive enrollment of second-trimester participants inherently limit its ability to establish causal relationships or capture longitudinal changes across the full gestational period. In a systematic review and meta-analysis of eight cross-sectional studies involving over 20,000 participants, Luo et al. (26) found that ID was significantly associated with elevated TSH levels, decreased FT4 levels, and a higher risk of clinical or subclinical hypothyroidism in pregnant women. While this represents the largest pooled dataset currently available on the topic, the evidence is entirely derived from cross-sectional designs. Most critically, the analysis completely failed to adjust for or stratify by iodine nutritional status. In a cross-sectional study enrolling 1,900 first-trimester pregnant women, Veltri et al. (80) confirmed ID as an independent risk factor for thyroid autoimmunity even after multivariate adjustment. However, the study lacked assessment or adjustment for individual iodine status; conducted in a mildly iodine-deficient region, it cannot rule out confounding from inadequate iodine nutrition. Furthermore, its exclusive focus on early pregnancy precludes capture of the dynamic interplay between Fe status and thyroid autoimmunity across full gestation. This between-study heterogeneity is driven primarily by variations in study populations, enrollment gestational windows, and ID diagnostic criteria, alongside inconsistent adjustment for iodine status.Future research requires large, prospective cohort studies with rigorous individual-level iodine control to validate these findings. Clinically, gestational iron status assessment should be paired with routine iodine monitoring to accurately stratify individual thyroid dysfunction risk.

Animal studies have further confirmed that maternal ID disrupts offspring thyroid function. In a perinatal dietary intervention rat model, Bastian et al. (81) first demonstrated that ID from early pregnancy through lactation significantly reduces TH levels in neonatal rat circulation and brain, alongside altered expression of cerebral TH-responsive genes, providing direct mechanistic evidence for the link between gestational ID and impaired offspring thyroid function. However, this work was limited to a rat model, with no stratification by iodine nutritional status or assessment of iodine-iron interactive effects, restricting its generalizability to human clinical settings.

## 4 Zinc and thyroid diseases

Zn is a crucial component of over 1,000 proteins, including transcription factors, enzymes, transporters, and receptors. Consequently, Zn plays a significant role in various biological processes such as growth and development, immune function, gene expression, reproduction, wound healing, redox regulation and apoptosis, and may affect thyroid function (9–11). In contrast to Fe, which can be accumulated and stored within the body, Zn necessitates regular dietary intake due to the absence of specific mechanisms for its long-term storage in humans. Up to now, Zn status *in vivo* has been assessed mainly through serum/plasma Zn concentration, hair Zn concentration, and urinary Zn excretion (11). Zn deficiency in patients with thyroid diseases is often underrecognized, so it is necessary to assess whether Zn deficiency exists. Zn supplementation can help prevent or treat thyroid diseases.

### 4.1 Zinc and hypothyroidism

Zn deficiency can lead to hypothyroidism (82). Zn-deficient diets and low serum Zn concentrations have been reported to affect THs metabolism by affecting the HPT axis (83, 84), thyronine deiodinase activity (85), binding of T3 to its nuclear receptor (86), and synthesis of thyroid transcription factor 2 (87) (Figures 1, 2). Moreover, Zn deficiency has been associated with structural modifications and volumetric alterations in thyroid tissue (88–90). In addition, Zn deficiency can potentially have an indirect impact on the status of THs by reducing energy intake (90).

#### 4.1.1 Clinical studies of zinc supplementation in treatment of hypothyroidism

Human studies have confirmed that Zn deficiency is significantly associated with abnormalities in THs in adult patients with overt hypothyroidism. Subclinical hypothyroidism stands as the most prevalent endocrine disorder among patients diagnosed with Down syndrome. Moreover, these patients these patients often have Zn deficiency. A systematic review and meta-analysis of 32 observational studies demonstrated that, compared with healthy controls, adult patients with overt hypothyroidism had significantly lower serum Zn concentrations, particularly in those with non-autoimmune hypothyroidism; whereas no significant difference in serum Zn concentrations was detected in adult patients with subclinical hypothyroidism (47). Targeted Zn supplementation modulates TH metabolism and alleviates hypothyroidism-associated manifestations in a population-specific manner. In a prospective interventional study, daily supplementation of 30 mg elemental Zn for 6 months was administered to patients aged 16–30 years with goiter concurrent with hypothyroidism and confirmed Zn deficiency,this intervention significantly elevated serum FT3 and FT4 concentrations, reduced TSH levels, and corrected Zn deficiency (91). In another randomized double-blind placebo-controlled trial, daily supplementation with 30 mg of elemental Zn alone for 12 weeks was administered to female patients with hypothyroidism aged 25–65 years, who were overweight or obese and receiving stable L-T4 replacement therapy (92). This regimen significantly elevated serum FT3 concentrations and the FT3/FT4 ratio (92). Furthermore, a randomized double-blind placebo-controlled trial demonstrated that 10 weeks of combined supplementation with Zn, vitamin A and magnesium markedly raised serum FT4 concentrations and alleviated chronic low-grade inflammation in patients with hypothyroidism aged 20–65 years undergoing L-T4 replacement therapy (93). For patients with hypothyroidism presenting with persistent symptoms such as alopecia, depressive states, decreased appetite, and skin lesions—who show an inadequate response to L-T4 monotherapy—case reports have demonstrated that Zn supplementation can reverse these clinical manifestations and enhance the response to TH replacement therapy (94). Therefore, targeted Zn supplementation can improve thyroid function-related biochemical parameters and hypothyroidism-related clinical manifestations in specific populations, namely adult patients with overt hypothyroidism and coexisting Zn deficiency, and patients with inadequate clinical response to L-T4 monotherapy.

#### 4.1.2 Effect of zinc deficiency on HPT axis

It is well known that Zn plays a regulatory role in the production of TRH within hypothalamic neurons and participates in the generation of TSH in pituitary cells (90, 95) (Figure 1). A rat study demonstrated that Zn serves as a cofactor of carboxypeptidase, which converts prepro-TRH into TRH through post-translational processing (96). Pyroglutamyl aminopeptidase II, a metallopeptidase reliant on Zn, is responsible for degrading the TRH secreted by the hypothalamus. Moreover, it engages in the function of the HPT axis by regulating the release of TSH from the adenohypophysis induced by TRH. Alvarez-Salas et al. (97) demonstrated that Zn deficiency in rats can cause the down-regulation of pyroglutamyl aminopeptidase II, resulting in elevated TSH levels, and leading to the occurrence of subclinical hypothyroidism. A human interventional study found that serum TSH, T4 and FT4 showed a downward trend during the period of low Zn diet, but an upward trend during a Zn-sufficient diet (84). However, only the decrease in FT4 was significant, and this downward trend of TSH, T4 and FT4 may be secondary to the decrease of TRH concentration, or it may be caused by the weakened response of TSH to TRH stimulation, as demonstrated in a rat study by Morley et al. (83). Arreola et al. (98) discovered that administering Zn supplementation to patients suffering from chronic renal failure can produce a central influence, manifested as increased plasma TSH concentration. The underlying mechanism for this phenomenon might be attributed to the impact of Zn on the pituitary gland or could be a secondary consequence of the augmented secretion of TRH from the hypothalamus (98). So Zn deficiency may participate in the biosynthesis or release of HPT axis hormone in patients suffering from chronic renal failure.

#### 4.1.3 Effect of zinc deficiency on the activity of iodothyronine deiodinase

Zn remains a necessary component of iodothyronine deiodinase, wherein D1 and D2 are key in facilitating the peripheral transformation of T4 into its biologically active T3 form (92). However, conflicting results have emerged regarding the effect of Zn deficiency on the transformation of T4 to T3. Morley and colleagues discovered that, when compared with paired calorie - restricted rats, only T3 levels were notably decreased in Zn-deficient rats (83). This phenomenon was ascribed to the diminished conversion rate of T4 to T3. Therefore, Zn is a necessary condition for the conversion of T4 to T3 outside the thyroid. Moreover, the maintenance of the serum T3 level might depend on Zn. Fujimato et al. (99) confirmed this view in a rat model, proposing that Zn was of equal significance to selenium or iodine in maintaining the homeostasis of THs. In a separate study, it was discovered that rats with Zn deficiency exhibited lower serum concentrations of T3 and FT4 (85). Additionally, the activity of hepatic D1 was diminished in these rats (85). Maxwell et al. (100) found that Zn supplementation could increase THs level (especially T3 level) in Zn deficient patients, which may be due to the increased activity of iodothyronine deiodinase. However, in another study, Zn deficiency instigated a reduction in serum T4 levels in subjects, whereas no corresponding decrease was observed in T3 levels (84), which was suggested to be ascribed to the heightened conversion efficiency of T4 to T3. The effect of Zn deficiency on iodothyronine deiodinase activity still needs more research.

#### 4.1.4 Other mechanisms

Zn participates in the process of T3 binding to its nuclear receptor (101). The function of T3 in target tissues is initiated by binding to a specific receptor (a nuclear receptor protein containing a Zn finger domain) located within the nucleus. This process requires Zn ions to form its biologically active conformation; therefore, Zn deficiency impairs the signal transduction of THs (86) (Figure 2B). Notably, GLI-Similar 3, one of the transcription factors containing a Zn finger domain, regulates the transcription of TH biosynthetic and TSH-inducible genes (such as sodium/iodide symporter, TPO, and TG) in thyroid follicular cells (102). Thus, mutations in the GLI-Similar 3 gene lead to congenital neonatal hypothyroidism (102). Thyroid transcription factor 2 is a Zn finger protein that regulates the expression of TG and TPO genes, and this regulatory process necessitates the involvement of Zn ions (87). Both *in vitro* and animal model studies mentioned above indicate that Zn is essential for THs synthesis.

Zn deficiency can result in alterations to both the structure and the volume of the thyroid gland. It has been reported that a Zn-deficient diet and a low concentration of Zn in the serum can induce structural alterations in the thyroid gland of guinea pigs, which are manifested as smaller thyroid volume, atrophy and degeneration of thyroid follicles (89). Ruz and colleagues demonstrated in a rat model that Zn deficiency is capable of inducing significant structural modifications and apoptosis in the thyroid follicular cells (90).

### 4.2 Zinc and AITD

Zn has been recognized for its potential benefits in immune regulation, anti-oxidative stress and anti-inflammatory, and Zn deficiency can increase autoimmune susceptibility (5). Zn can regulate cell function and signal transduction, and all immune cells are affected by Zn signals (11). It can promote T cell proliferation and differentiation, and induce the synthesis of cytokines such as IL-1, IL-6, and TNF-α, thereby enhancing the synergistic effects between immune cells (103). Immune cells may respond to Zn deficiency more quickly than measurable Zn in plasma (104). Zn is key to thymic function, which provides a unique microenvironment for naive T cell proliferation, differentiation, and maturation (105). The Zn-binding thymic factor (thymosin), synthesized by thymic epithelial cells, needs Zn for bioactivity induction. Upon thymosin activation of thymic epithelial cells, positive selection screens T cells that bind self- main histocompatibility complex and recognize foreign antigens (105). Zn participates in or regulates the activity of multiple DNA repair proteins, which are crucial for maintaining the integrity of DNA (106). Additionally, Zn can increase the activities of antioxidant proteins and antioxidant enzymes, such as glutathione, catalase and superoxide dismutase (SOD), and increase the gene expression of Zn finger proteins with anti-inflammatory effects, such as A20 and PPAR-a (104, 107). Zn deficiency has been shown to increase oxidative stress and inflammatory cytokines. Therefore, Zn deficiency may contribute significantly to the pathogenesis of AITD.

The quality of current clinical evidence on Zn and AITD risk varies considerably. Two key studies have failed to establish a significant association between Zn status and AITD, though both are hindered by distinct methodological flaws. For instance, Przybylik-Mazurek et al. (108) conducted a small cross-sectional study (*n* = 37) revealing no divergence in serum Zn between female HT patients and healthy controls; however, this finding is fundamentally compromised by an exceptionally tiny cohort that yields inadequate statistical power. The second, a sizable single-center study by Moncayo et al. (109) (*n* = 1,401), similarly failed to detect any correlation between serum Zn and AITD onset, thyroid function, or autoantibody titers. Despite rigorous quality controls in trace element profiling, its overall validity is substantially diluted by a low proportion of AITD cases, the omission of subgroup analyses stratified by thyroid function, and the inherent limitation that serum Zn alone offers an incomplete picture of systemic Zn metabolism. Conversely, multiple studies have identified significant associations between Zn dyshomeostasis and AITD risk, with graded levels of evidence quality. Ertek et al. (88) (*n* = 201) reported a positive correlation between serum Zn and thyroid autoantibody titers in AITD patients, with iodine deficiency confounding controlled, but was limited by a single-region Turkish cohort and cross-sectional design unable to confirm causality. Stojsavljević et al. (110) (*n* = 103) quantified Zn levels across three sample matrices—thyroid tissue, whole blood, and urine—and reported significantly reduced whole-blood Zn in patients with histopathologically confirmed HT, with no significant between-group differences in thyroid tissue or urinary Zn levels. However, this study carried severe selection bias, as it exclusively enrolled HT patients requiring surgical intervention, and the cohort was limited to women from a single geographic region. The most methodologically robust, highest-grade evidence originates from a large 1:1 matched case-control study by Vargas-Uricoechea et al. (111) (*n* = 1,048). This work rigorously adjusted for key confounders linked to Zn metabolism, and demonstrated a robust association between reduced serum Zn concentrations and elevated AITD risk, particularly in individuals with serum Zn < 70 μg/dL. This between-study heterogeneity arises primarily from four core sources: variation in Zn measurement matrices, disparities in study design, variability in baseline population traits (including iodine nutritional status and sex distribution), and differing rigor in the adjustment for confounding variables. Taken together, available evidence supports a potential link between Zn deficiency and elevated AITD risk, yet nearly all data are derived from observational studies. Future multicenter, prospective cohort studies employing standardized Zn testing methodologies are warranted to definitively establish causal relationships and define clinically relevant cutoff values.

### 4.3 Zinc and thyroid cancer

Zn exerts anti-cancer defensive effects via multiple pathways, including immune regulation, transcription factor modulation, antioxidant defense, and maintenance of genomic stability (112). Chronic Zn deficiency markedly elevates the risk of cancer development, and Zn functions in a strictly dose-dependent manner: Zn at physiological levels boosts immunity and exerts tumor-suppressive effects, while excessive supplementation carries potential risks such as immunosuppression (112, 113). Because the immune system serves as the body's primary defense against cancer, and Zn is indispensable for immune cell development, activation, and function, systemic depletion directly blunts anti-tumor immune responses (114). Furthermore, persistent activation of the NF-κB pathway is heavily implicated in tumor proliferation, angiogenesis, metastasis, chemoresistance, and the evasion of apoptosis; Zn counteracts these processes by negatively regulating NF-κB via several distinct mechanisms (115–117). Addressing oxidative stress—a core driver of tumorigenesis—Zn acts as a crucial cofactor for SOD1. By shielding cells from ROS-inducing agents, SOD prevents cancer initiation and progression (118).Zn selectively protects normal cells against the cytotoxic and genotoxic damage induced by H_2_O_2_, while enhancing the lethal effect of H_2_O_2_ on tumor cells (119). In addition, Zn plays a critical role in DNA replication, repair, and transcriptional regulation by stabilizing Zn finger structures. Zn deficiency can lead to disruption of genomic integrity, impaired DNA repair efficiency, and a significantly elevated risk of carcinogenesis (106). It must be explicitly noted that the mechanistic evidence underpinning zinc's anti-tumor effects is almost exclusively drawn from preclinical *in vitro* and *in vivo* animal studies of other tumor types. To date, targeted validation in thyroid cancer models remains remarkably scarce.

Changes in Zn concentrations in serum and thyroid tissue may be related to the pathogenesis of thyroid cancer, and several observational studies have shown that serum Zn levels are reduced in patients with thyroid cancer. Emami et al. (120) and Kazi et al. (121) conducted studies which demonstrated that the serum Zn concentration in patients with medullary thyroid carcinoma and PTC was lower than that of healthy people. Al-Sayer et al. (122) found that serum Zn levels were lower in patients with thyroid carcinoma as compared to normal subjects, whereas serum Zn levels were significantly higher after surgical excision of cancerous tissues, which may be due to the fact that removal of the tumors eliminated the flow of Zn into the cancerous tissues. The study of Baltaci et al. (95) reinforced this view. They found that serum Zn levels in patients with PTC were lower in both the preoperative and postoperative periods, and returned to normal values by 15 days postoperatively. However, Zn levels in the thyroid tissues were higher than those of controls. Therefore, the serum Zn level may have an impact on the long-term follow-up of patients with thyroid carcinoma, and the change of Zn concentration in serum and thyroid tissue may be related to the pathogenesis of thyroid carcinoma. While assessing Zn deficiency is essential for optimizing tailored nutritional therapies in thyroid cancer patients, the optimal prophylactic dose for cancer prevention remains undetermined.

Taken together, research on Zn and thyroid cancer shows a stark translational disconnect between preclinical findings and clinical evidence. Future work must center on thyroid cancer-specific preclinical experiments, large prospective clinical studies, and interventional trials to validate these preclinical mechanisms.

## 5 Copper and thyroid diseases

Cu acts as a cofactor for upwards of 30 enzymes, among which are cytochrome C oxidase, SOD, and lysyl oxidase. Cu can be biotransformed between different redox states, namely oxidation state [cupric (II)] and reduction state [cuprous (I)] as a transition metal (123). Therefore, Cu is indispensable for important physiological processes such as energy metabolism, antioxidant metabolism, coagulation and signal transduction in the body (10). Most of the Cu in the body is stored in the liver, so the liver is a key tissue in maintaining Cu homeostasis (10). 95% of the Cu in plasma is bound to ceruloplasmin, which carries Cu through tissues and blood to play a role (124). On the other hand, Cu also has potential harmful effects, and cuprous (I) can produce hydroxyl radicals in Fenton reaction with hydrogen peroxide, thereby causing damage to lipids, proteins and DNA (125). In addition, Cu can also replace Fe atoms in Fe-sulfur clusters, inactivating various enzymes containing Fe-sulfur clusters (126). Therefore, it is crucial to maintain the balance of Cu in the body.

### 5.1 Copper and hypothyroidism

#### 5.1.1 Clinical study on the relationship between copper and hypothyroidism

A cross-sectional investigation conducted by Blasig and colleagues revealed a notable positive association between the serum Cu and THs in children with congenital hypothyroidism (127). Therefore, thyroid axis in these children is closely related to Cu metabolism. Furthermore, it was also found that serum Cu levels in rats with hypothyroidism induced by methimazole were notably lower compared to those in the control group (128). Razaei and co – workers (129) discovered that a significant reduction in Cu levels would lead to hypothyroidism. In contrast, Baltaci et al. (130) discovered that hypothyroidism was linked to relatively high Cu levels. Hanif et al. (131) and Stojsavljevc et al. (132) found that the serum Cu level in hypothyroidism patients was significantly higher compared to that in healthy controls. In addition, a meta - analysis have indicated that there is no substantial disparity in the blood Cu concentrations between individuals with hypothyroidism and healthy control participants (47).

#### 5.1.2 Mechanisms of copper deficiency affecting hypothyroidism

Reports have indicated that Cu deficiency can exert an influence on the metabolism of THs via the following pathways (Figures 1, 2). A rat model study found that Cu deficiency may disrupt central control of the HPT axis (81) (Figure 1). In an *in vitro* study, Cu deficiency was found to regulate the activity of TRH by affecting the activity of cuproenzyme peptidyl-glycine-α-amidating monooxygenase (133). This enzyme participates in the biosynthesis of α-amidating peptides, with TRH being among them, and demands two Cu atoms to achieve optimal amidation activity (133). Consequently, a decrease in the Cu content within the hypothalamus might result in a decline in the activity of TRH. In contrast to ID rats, pituitary TSH is normally released into serum after intravenous administration of exogenous TRH in adult Cu deficiency rats. Therefore, it can be concluded that Cu deficiency does not attenuate the TSH response to TRH (134). In one study, serum total-T3 levels were 48% lower, serum total-T4 levels were 21% lower, and whole-brain T3 levels were 10% lower in 12-day-old offspring of Cu deficiency pregnant rats compared to the offspring of Cu-normal pregnant rats (81). Analysis of brain mRNA showed that the expression of several TH-response genes, such as small albumin, myelin basic protein and early growth response factor 1, were altered in Cu deficiency offspring pups, suggesting that the brain of the Cu deficiency neonates sensed the reduced THs concentration (81). These findings imply that the brain defects related to neonatal Cu deficiency are mediated, at least in part, by reduced circulating and cerebral TH levels. Animal studies have shown that Cu deficiency can reduce the activity of D1 in liver (135) and D2 in brown adipose tissue (136), thus affecting the peripheral and local T4 to T3 conversion (Figure 2). Cu is an indispensable element in the process of tyrosine metabolism, and the production of THs is inseparable from tyrosine (137) (Figure 1). Lukaski et al. (136) observed that in mice with Cu deficiency, the circulating concentrations of T3 and T4 were reduced, but the T4/T3 ratio was not affected, which may be because the decreased production and release of T4 in the thyroid and the decrease of deiodinase (D1 and D2) affecting the conversion of T4 to T3, thus keeping the T4/T3 ratio relatively constant. It was discovered that T3 binding to nuclear receptors was inversely correlated with Cu status, possibly indicating cellular adaptation to hypothyroidism (136). Wu et al. (138) observed a positive correlation between the levels of Cu in the whole blood of pregnant women during the second trimester and the elevation of the FT3/FT4 ratio in a human observational study. This finding suggests that Cu levels could potentially play a significant role in the transformation of FT4 into FT3 throughout pregnancy. In addition, SOD serves as the principal enzyme within the intracellular antioxidant defense system, and Cu is an important cofactor of this enzyme, therefore, Cu deficiency in humans may increase oxidative stress in thyroid cells, which may affect THs synthesis (139).

### 5.2 Copper and AITD

Cu is involved in the function of immune cells such as T cells, B cells, neutrophils, natural killer cells and macrophages, and Cu deficiency can affect innate and adaptive immunity (140). The ability of peripheral blood neutrophils to produce superoxide anion and kill microorganisms, as well as the number of neutrophils, are decreased in the presence of Cu deficiency (141). Disruption of serum Cu homeostasis may lead to a state of oxidative stress in HT patients (142). SOD is the primary enzyme in the cellular antioxidant defense system, responsible for removing oxygen free radicals and preventing oxidative stress damage. Cu and Zn serve as the main cofactors of SOD1, and the role of Zn ion is mainly to maintain the secondary structure of the enzyme, while the role of Cu ion is mainly to maintain the activity of the enzyme. The Cu content of SOD accounts for about 60% of the total Cu in human erythrocytes (118, 143). Changes in the activity of this enzyme are consistent with serum levels of Zn and Cu (122). Cu acts as an antioxidant, eliminating free radicals and reducing cellular damage during TH metabolism (144). Oxygen free radicals may be involved in the process of postocular fibroblast proliferation in patients with Graves' ophthalmopathy (145). A study investigated the levels of serum trace elements in patients with GD in areas of northeastern China with adequate iodine intake. and found that higher serum Cu levels may be associated with thyroid autoimmunity, as evidenced by relatively high serum Cu levels in patients with GD and low serum Cu levels in patients with GD combined with Graves' ophthalmopathy (8). A recent study conducted by Rasic-Milutinovic et al. (142) discovered that the blood Cu concentration in HT patients was significantly higher than that in healthy people, and the Cu/Se ratio was independently correlated with TH level in HT patients, indicating that the Cu/Se ratio may affect thyroid function in HT patients directly. In contrast, Stojsavljević et al. (110) reported a negative correlation between the risk of AITD and serum Cu levels. However, some studies have also concluded that there is no significant difference in serum Cu levels and Cu/Zn ratio between HT and healthy individuals (48, 108). In view of this, to clarify the pathogenic mechanisms of Cu in AITD, it is necessary to break through the existing limitations of observational studies, shift to hypothesis-driven experimental research, and conduct an in-depth analysis of its specific molecular mechanisms.

### 5.3 Copper and thyroid cancer

Cu imbalance is associated with cancer (146). It is well known that antioxidant defense systems play an important role in the development of benign and malignant tumors. Cu can act as an antioxidant and pro-oxidant. As an antioxidant, Cu has the ability to remove or neutralize free radicals, potentially reducing or preventing damage caused by them (147). As a pro-oxidant, high concentrations of Cu can be toxic to cells. It is well known that Wilson's disease is an inherited disorder with abnormal Cu metabolism. Additionally, high concentrations of Cu induce tumorigenesis through the production of toxic free hydroxyl radicals that damage DNA (148). Excessive production of ROS can lead to damage of DNA and proteins, and ultimately lead to cancer (146). Cu is a major cofactor of SOD1, and SOD prevents the occurrence and development of tumors by protecting cells from substances that cause ROS formation (118). However, the overexpression of SOD presents a potential risk for carcinogenesis (125).

The primary pathway for ATP production in mammals is through oxidative phosphorylation in mitochondria, with the Cu-binding enzyme cytochrome c oxidase playing a crucial role in this process. The secondary pathway is glycolysis. Ishida et al. (146) discovered that different levels of Cu intake could regulate the activity of cytochrome c oxidase in cancer cells, thus affecting the level of mitochondrial oxidative phosphorylation. Inhibition of systemic Cu by Cu chelators hinders oxidative phosphorylation, leading to a compensatory increase in glycolysis for Cu-deficient cancer cells, yet overall ATP levels remain reduced (146). This suggests that Cu can serve as a rate-limiting nutrient for tumors, similar to oxygen and glucose. Therefore, Cu has the potential to be utilized for targeted tumor therapy.

Cu is an essential cofactor in tumor angiogenesis, and serum Cu content is abnormally high in many types of progressive tumors (149). Additionally, some Cu-binding molecules such as plasma ceruloplasmin, heparin and the Glycyl histidine tripeptide promote angiogenesis only after binding with Cu (149). Cu chelators have anti-angiogenic activity (150). However, the specific mechanism of Cu in angiogenesis has not been fully clarified.

A meta-analysis revealed that serum Cu levels were higher in patients with thyroid cancer than in healthy controls, especially in Chinese thyroid cancer patients (144). Most clinical observational studies have concluded an increase in serum Cu and a decrease in serum Zn levels in malignant tumors, especially in thyroid cancer (95, 118, 143). For patients with tumors, the Cu/Zn concentration ratio is a more sensitive indicator than a single concentration of these metals (151). The homeostatic balance between Zn and Cu has been well documented in preclinical studies. It is known that a high dose of Zn in the diet can inhibit the absorption of Cu in the intestine and the accumulation of Cu in the liver, similarly, a high intake of Cu can also inhibit the absorption of Zn (10, 152). Competition for mineral-binding ligands and mineral uptake sites in the intestinal mucosa is considered a major factor contributing to Zn and Cu antagonism. High Zn intake induces increased expression of metallothionein in the intestinal mucosa, which exhibits a high binding affinity for Cu and can protect cells from damage caused by excessive metal accumulation in tissues (152). Dragutinovi et al. (143) conducted a retrospective study and concluded that the serum Cu ion concentration of patients could serve as a useful and simple tool for preoperative prediction of PTC in patients with thyroid diseases. Baltaci et al. (95) designed a case-control study and discovered that serum Cu levels were significantly higher in patients with PTC both preoperatively and postoperatively, and returned to normal values 15 days after surgery. The study also revealed a negative correlation between serum Zn and Cu levels, leading the authors to suggest that the increased Cu levels may be attributed to lower Zn levels (95). In a separate case-control study, Kucharzewski et al. (118) discovered that the ratio of Cu/Zn and Cu/Se concentrations in whole blood and thyroid tissue was higher in patients with PTC compared to patients with GD and nodular goiter (118). Therefore, Cu/Zn and Cu/Se may serve as superior biochemical indicators for the diagnosis of thyroid cancer. The clinical observation conducted by Kazi et al. (121) and Stojsavljevic et al. (153) further confirmed the finding that the plasma Cu/Zn ratio in patients with PTC was significantly higher compared to the control group. The conventional 65Cu/63Cu ratio (δ65Cu) is used to report the abundance of Cu isotopes. Kazi et al. (121) also discovered that blood δ65Cu was significantly lower in PTC patients compared to healthy controls, while δ65Cu was higher in thyroid tumors. Therefore, the Cu/Zn ratio and Cu isotope can serve as biomarkers for rapid screening and diagnosis of thyroid cancer.

Overall, the link between Cu and thyroid cancer carries a stark translational disconnect: proposed tumor-related mechanisms derive almost entirely from preclinical work without thyroid cancer-specific validation, while human data are limited to observational studies, with no large prospective cohorts to confirm causal links to Cu homeostasis dysregulation, nor interventional trials to validate Cu chelation therapy efficacy. Future research should prioritize filling these critical evidence gaps.

## 6 Conclusions

In this narrative review, we systematically synthesized the associations between Fe, Zn, Cu and multiple thyroid diseases, with all core findings and regulatory mechanisms comprehensively summarized in Table 1. Our analysis reveals a clear hierarchy of evidence robustness among the three metals: Fe has the most sufficient and clinically translatable evidence, with established adjunctive clinical value in hypothyroidism management; available data for Zn are largely restricted to hypothyroidism and AITD, with critical gaps in thyroid cancer and gestational populations; clinical findings for Cu are highly heterogeneous across all disease subtypes, with the most promising application potential as an auxiliary diagnostic biomarker for thyroid cancer.

**Table 1:** Summary of the main effects of essential metals (Fe, Zn, Cu) on thyroid diseases.

Essential metal	Thyroid disease category	Summary of clinical & preclinical evidence	Key regulatory mechanisms
**Fe**	Hypothyroidism	1. For adult patients with primary subclinical or overt hypothyroidism who have a serum ferritin level < 70 μg/L, or concurrent ID/IDA, combined L-T4 and Fe supplementation can effectively improve thyroid function. 2. Patients with β-thalassemia are prone to IO due to long-term blood transfusion, and this Fe-mediated toxic injury can induce primary hypothyroidism.	1. ID impairs THs synthesis and metabolism by inhibiting the HPT axis, reducing TPO activity, suppressing mitochondrial oxidative phosphorylation, disrupting deiodinase function, and blocking T3-nuclear receptor binding. 2. IO triggers thyroid cytotoxicity via elevated ROS, lipid peroxidation markers, and saturated/unsaturated aldehyde production.
	AITD	Clinical studies have shown reduced serum Fe in HT patients, and a negative correlation between serum Fe and TPOAb levels in women of childbearing age.	1. ID elevates AITD risk via impaired DNA synthesis, enhanced oxidative stress, broken immune tolerance, and cellular/humoral immune dysfunction. 2. IO dysregulates immune function, damages thyroid tissue, exposes intracellular antigens (TPO/TG), and stimulates thyroid autoantibody production.
	Thyroid cancer	Preclinical studies have validated two potential anti-tumor strategies for thyroid cancer: inhibiting tumor growth via Fe deprivation (cancer cell Fe depletion), and killing cancer cells through ferroptosis (Fe-driven cytotoxic oxidative stress in tumor cells).	1. Fe acts as a potential carcinogenic mutagen: it catalyzes free radical generation via the Fenton reaction, causing DNA strand breaks, protein inactivation, and lipid peroxidation. 2. Targeting Fe metabolism-related proteins (transferrin, ferroportin, Fe responsive element-binding proteins) suppresses tumor growth.
	Gestational thyroid dysfunction (maternal and offspring)	1. Clinical studies have linked gestational ID to higher risks of maternal thyroid dysfunction and AITD. 2. Animal studies confirmed maternal ID disrupts offspring thyroid function and alters TH-responsive gene expression in the offspring's brain.	–
**Zn**	Hypothyroidism	Clinical studies have shown targeted Zn supplementation improves thyroid function-related biochemistry and hypothyroid symptoms in specific populations: adults with overt hypothyroidism and concurrent Zn deficiency, and patients with poor clinical response to L-T4 monotherapy.	ZnD impairs THs synthesis and metabolism by inhibiting the HPT axis, blocking T3-nuclear receptor binding, and impairing the function of transcription factors (TTF-2 and GLI-Similar 3). It is also associated with structural and volumetric changes in thyroid tissue.
	AITD	Clinical studies have found reduced serum Zn is significantly associated with higher AITD onset risk, with notably higher AITD prevalence in populations with serum Zn < 70 μg/dL.	Zn deficiency can compromise immune function, impaire DNA repair capacity, and enhance oxidative stress and inflammatory responses.
	Thyroid cancer	1. Clinical studies showed significantly reduced serum Zn in thyroid cancer patients, which returned to normal levels after surgical tumor resection. 2. Zn levels were higher in thyroid cancer tissues than in paired adjacent normal tissues.	Zn exerts anti-tumor effects via immune regulation, antioxidant defense, negative modulation of the NF-κB pathway, and maintenance of genomic stability.
**Cu**	Hypothyroidism	Existing clinical findings have been highly heterogeneous. Some studies reported significantly lower serum Cu in hypothyroid patients vs. healthy controls.	Cu deficiency blocks TH synthesis and metabolism by inhibiting the HPT axis, disrupting deiodinase function, suppressing mitochondrial oxidative phosphorylation, impairing tyrosine metabolism, and exacerbating oxidative stress in thyroid cells.
	AITD	Existing observational studies have high heterogeneity: some showed elevated serum Cu in AITD patients, while others reported a negative correlation between serum Cu and AITD risk.	Dysregulated Cu homeostasis causes immune cell dysfunction, oxidative stress imbalance, and impaired ROS clearance, which may induce thyroid autoimmune injury.
	Thyroid cancer	1. Clinical studies found significantly higher serum Cu in thyroid cancer patients vs. healthy controls. 2. Cu/Zn ratio and Cu isotopes can serve as potential biomarkers for rapid screening of PTC.	1. High Cu levels generate toxic hydroxyl radicals via the Fenton reaction, causing DNA/protein damage and driving carcinogenesis. 2. Physiological Cu exerts antioxidant effects. 3. Cu chelators have anti-tumor effects via inhibiting angiogenesis and blocking oxidative phosphorylation.

Critically, most existing mechanistic evidence comes from preclinical models and cannot be directly extrapolated to human clinical settings. Clinical data are mainly from observational studies, with a clear lack of large prospective randomized controlled trials. The current research landscape is severely imbalanced: high-quality studies on Zn and Cu are still scarce compared with Fe, especially in gestational cohorts.

Overall, homeostasis disturbances of these essential metals can adversely affect thyroid function, and their clinical value in thyroid disease management has been significantly underestimated. Future research should prioritize a bench-to-bedside translational framework to validate causal associations and establish standardized clinical strategies for trace element management in thyroid care.
